# Ultra fast, accurate PET image reconstruction for the Siemens hybrid MR/BrainPET scanner using raw LOR data

**DOI:** 10.1186/2197-7364-2-S1-A37

**Published:** 2015-05-18

**Authors:** Juergen Scheins, Christoph Lerche, Jon Shah

**Affiliations:** Forschungszentrum Jülich GmbH, Jülich, Germany

Fast PET image reconstruction algorithms usually use a Line-of-Response (LOR) preprocessing step where the detected raw LOR data are interpolated either to evenly spaced sinogram projection bins or alternatively to a generic projection space as for example proposed by the PET Reconstruction Software Toolkit (PRESTO) [1]. In this way, speed-optimised, versatile geometrical projectors can be implemented for iterative image reconstruction independent of the underlying scanner geometry. However, all strategies of projection data interpolation unavoidably lead to a loss of original information and result in some degradation of image quality. Here, direct LOR reconstructions overcome this evident drawback at cost of a massively enhanced computational burden. Therefore, computational optimisation techniques are essential to make such demanding approaches attractive and economical for widespread usage in the clinical environment. In this paper, we demonstrate for the Siemens Hybrid MR/BrainPET with 240 million physical LORs that a very fast quantitative direct LOR reconstruction can be realized using a modified version of PRESTO. Now, PRESTO is also capable to directly use sets of symmetric physical LORs instead of interpolating LORs to a generic projection space. Exploiting basic scanner symmetries together with the technique of Single Instruction Multipe Data (SIMD) and Simultaneous Multi-Threading (SMT) results in an overall calculation time of 2-3 minutes per frame on a single multi-core machine, i.e. neither requiring a cluster of mutliple machines nor Graphics Processing Units (GPUs).

